# Attenuation of Inflammatory Mediators (TNF-α and Nitric Oxide) and Up-Regulation of IL-10 by Wild and Domesticated Basidiocarps of *Amauroderma rugosum* (Blume & T. Nees) Torrend in LPS-Stimulated RAW264.7 Cells

**DOI:** 10.1371/journal.pone.0139593

**Published:** 2015-10-01

**Authors:** Pui-Mun Chan, Yee-Shin Tan, Kek-Heng Chua, Vikineswary Sabaratnam, Umah Rani Kuppusamy

**Affiliations:** 1 Mushroom Research Centre, University of Malaya, 50603, Kuala Lumpur, Malaysia; 2 Institute of Biological Sciences, Faculty of Science, University of Malaya, 50603, Kuala Lumpur, Malaysia; 3 Department of Biomedical Science, Faculty of Medicine, University of Malaya, 50603, Kuala Lumpur, Malaysia; University of Kentucky, UNITED STATES

## Abstract

*Amauroderma rugosum*, commonly known as “Jiǎzī” in China, is a wild mushroom traditionally used by the Chinese to reduce inflammation, to treat diuretic and upset stomach, and to prevent cancer. It is also used by the indigenous communities in Malaysia to prevent epileptic episodes and incessant crying by babies. The aim of this study was to compare the wild and domesticated basidiocarps of *A*. *rugosum* for antioxidant and *in vitro* anti-inflammatory effects in LPS-stimulated RAW264.7 cells. The wild basidiocarps of *A*. *rugosum* were collected from the Belum Forest, Perak, Malaysia and the domesticated basidiocarps of *A*. *rugosum* were cultivated in the mushroom house located in the University of Malaya, Kuala Lumpur, Malaysia. Both the wild and domesticated basidiocarps were subjected to ethanolic extraction and the extracts were tested for antioxidant and anti-inflammatory activities. In this study, the crude ethanolic extract of wild (WB) and domesticated (DB) basidiocarps of *A*. *rugosum* had comparable total phenolic content and DPPH scavenging activity. However, WB (EC_50_ = 222.90 μg/mL) displayed a better ABTS cation radical scavenging activity than DB (EC_50_ = 469.60 μg/mL). Both WB and DB were able to scavenge nitric oxide (NO) radical and suppress the NO production in LPS-stimulated RAW264.7 cells and this effect was mediated through the down-regulation of inducible nitric oxide synthase (iNOS) gene. In addition, both WB and DB caused down-regulation of the inflammatory gene TNF-α and the up-regulation of the anti-inflammatory gene IL-10. There was no inhibitory effect of WB and DB on nuclear translocation of NF-κB p65. In conclusion, the wild and domesticated basidiocarps of *A*. *rugosum* possessed antioxidant and *in vitro* anti-inflammatory properties. WB and DB inhibited downstream inflammatory mediators (TNF-α and NO) and induced anti-inflammatory cytokine IL-10 production. No inhibitory effects shown on upstream nuclear translocation of NF-κB p65. WB and DB exhibited antioxidant activity and attenuation of proinflammatory mediators and therefore, *A*. *rugosum* may serve as a potential therapeutic agent in the management of inflammation.

## Introduction

Reactive oxygen species (ROS) are products of normal cellular metabolism. The products of partial reduction of oxygen such as superoxide anion radical (O^-2^), hydroperoxyl radical (HOO^·^) and hydroxyl radical (^·^OH) are highly reactive [1]. These reactive molecules play an important role in the defence against microbial pathogens [2]. However, overproduction of ROS can oxidise and damage normal functions of lipids, proteins and DNA [3]. Therefore, increased production of ROS is often associated with chronic inflammation and a wide variety of diseases [4].

Inflammation is a biological response of tissue in attempting self-protection against harmful stimuli such as pathogens, damaged cells and irritants. There are two types of inflammation; acute and chronic inflammation. Acute inflammation is often characterised in the four classical signs which are redness, heat, swelling, and loss of function. Monocytes extravasate from blood vessels into the injured site and transform into macrophages. Macrophages play a vital role in inflammatory response in the initiation, maintenance and resolution of inflammation. However, if the cause of inflammation cannot be eliminated, inflammation will prolong and vary in intensity over time [5]. Prolonged or chronic inflammation may contribute to detrimental outcomes such as chronic inflammatory diseases. Macrophages are activated by proinflammatory mediators such as lipopolysaccharide (LPS), interleukin-1β (IL-1β), and interferon-γ (IFN-γ) by binding to toll-like receptor-4 (TLR-4) which in turn activate the inflammatory signalling pathway nuclear factor kappa B (NF-κB) [6]. This subsequently stimulates the release of numerous proinflammatory mediators such as nitric oxide (NO), tumour necrosis factor-α (TNF-α), and interleukin-6 (IL-6) [6]. These proinflammatory mediators play a major role in the pathogenesis of various inflammatory disorders and serve as potent biomarkers for the assessment of the inflammatory process. Recent evidence also indicates that inflammation may be the main contributor to seizure generation and development of neurological disorders such as epilepsy [7]. Therefore, biomarkers of inflammation may serve as important molecular targets for the development of potential anti-inflammatory drugs and may be used to facilitate diagnosis and management of inflammatory disorders and neurologic diseases such as epilepsy [7 – 8].

Mushrooms have been used in traditional medicine for many centuries and they are well known for their medicinal properties. *Ganoderma lucidum* or commonly known as “Reshi”or “Lingzhi”, has a long history in traditional Chinese medicine for more than 4000 years and has been known to promote health and longetivity [9]. This popular medicinal mushroom has been reported to be used to prevent and treat various diseases such as chronic hepatitis, arthritis, hypertension, hyperlipedemia, asthma, diabetes and cancers [10]. *Ganoderma lucidum* belongs to the family *Ganodermataceae* and it is the most well studied member. *Ganodermataceae* comprises 15 genera and *Amauroderma* is one of them. *Amauroderma* is found in tropical areas and consists of about 30 species [11]. *Amauroderma rugosum* is a wild medicinal mushroom with a black stipe and a white-pored hymenium that bruises to a blood red colour when touched [12]. *Amauroderma* sp. is also known as the “epileptic child mushroom” or “cendawan budak sawan” in the Malay language and it is commonly known as “Jiǎzhī” in China. It is traditionally used by the Chinese to reduce inflammation, to treat diuretic and upset stomach, and to prevent cancer [13]. In Malaysia, the fresh stipe is diced, strung, and worn as a necklace by the indigenous Temuan people to prevent epileptic episodes and incessant crying by babies [14–15]. Volatile components which may be present in the mushroom, may have contributed to the beneficial effects of this mushroom. In our previous study, we have reported that the mycelium of *A*. *rugosum* grown in submerged culture is a good source of nutrients and the hexane fraction (ethyl linoleate and ergosterol) has potential antioxidant and anti-inflammatory activities [16]. However, the potential use of the basidiocarps of *A*. *rugosum*, bothwild and domesticatedin the mitigation of inflammation and the mode of action as well as the antioxidant properties are not known. Hence in this study, the wild and domesticated basidiocarps of *A*. *rugosum* were investigated for antioxidant activities and *in vitro* anti-inflammatory effects as well as the mode of action on LPS-stimulated RAW264.7 cells.

## Materials & Methods

### Chemicals

Potato dextrose agar (PDA) and potato dextrose broth (PDB) were purchased from Difco (BD, USA). Calcium carbonate (CaCO_3_) and ethanol were purchased from Systerm (Selangor, Malaysia). Dimethyl sulfoxide (DMSO) was purchased from Fisher Scientific Inc. (New Hampshire, USA) and 2, 2-Diphenyl-1-picrylhydrazyl (DPPH), ascorbic acid, trolox, butylated hydroxytoluene (BHT), gallic acid, Dulbecco’s Modified Eagle’s Medium (DMEM), foetal bovine serum (FBS), *Escherichia coli* (O55:B5) lipopolysaccharide (LPS), N_ω_-nitro-l-arginine-methyl ester (L-NAME), sulphanilamide, N-1(1-napthyl)ethylenediamine, phosphoric acid (H_3_PO_4_), quercetin, triton X-100, and sodium nitroprusside were obtained from Sigma-Aldrich (St. Louis, MO, USA). 2,2’-Azino-bis(3-ethylbenzothiazoline-6-sulphonic acid) (ABTS) and 3-(4,5-dimethylthiazol-2-yl)-2,5-diphenyltetrazolium bromide (MTT) were purchased from Calbiochem, Merck Millipore (Darmstadt, Germany). Potassium persulphate, Folin-Ciocalteau phenol reagent, and sodium carbonate (Na_2_CO_3_) were obtained from Merck & Co. (New Jersey, USA). Penicillin-streptomycin and fungizone were purchased from Biowest (MO, USA) and phosphate buffer saline (PBS) was obtained from Oxoid Ltd, Thermo Scientific (Hampshire, UK).

### Ethics statement

The wild basidiocarps (KLU-M 1369) of *A*. *rugosum* were collected from the Royal Belum Rainforest Reserve, Perak, Malaysia (N05°28’58.80 E101°20’24.72) with the permission from Pulau Banding Foundation, Perak, Malaysia during the 2^nd^ Temengor Scientific Expedition 2012. The specimens are not endangered or protected species.

### Mushroom collection and cultivation

The wild basidiocarps (KLU-M 1369) of *A*. *rugosum* were collected from the Royal Belum Rainforest Reserve, Perak, Malaysia (N05°28’58.80 E101°20’24.72) and authenticate identified by Mushroom Research Centre, University of Malaya, Kuala Lumpur, Malaysia, through morphology and molecular studies. The mycelium pure culture of *A*. *rugosum* was obtained by tissue culture from the fresh basidiocarp. Mycelia of *A*. *rugosum* was maintained on PDA medium as described in the previous study [16]. Mushroom cultivation of *A*. *rugosum* was done according to Moonmoon et al. [17] with slight modifications. The wheat grain was cleaned and soaked in tap water overnight. Thereafter, the soaked grains were drained and autoclaved at 121°C for 15 minutes. The sterilised grains were allowed to cool for 24 hours before inoculating with mycelia culture of *A*. *rugosum* and were then incubated at room temperature for 14 days until the mycelium completely covered the grains. Each mushroom bag contained 300 g of substrate comprising CaCO_3_ (1%), rice bran (10%), rubberwood saw dust (89%) and water (70%). The bags filled with the substrates were autoclaved at 121°C for 15 minutes to sterilise them. Then, the sterilised bags were left to cool for 24 hours before inoculating them with the prepared spawn. The bags were stored in the mushroom house (University of Malaya, Kuala Lumpur, Malaysia) under indirect sun light at 26°C with 80 to 85% relative humidity.

### Preparation of *A*. *rugosum* extracts

The wild and domesticated basidiocarps of *A*. *rugosum* were freeze dried and extracted with ethanol at a ratio of 1:10 (w/v) for two days at room temperature. The ethanolic extracts of the wild (WB) and domesticated (DB) basidiocarps of *A*. *rugosum* were decanted and filtered using Whatman No. 4 filter paper. The extraction process was repeated five times with ethanol at a ratio of 1:10 (w/v), the filtrates were combined, and the excess solvent was evaporated using rotary evaporator.

### Anti-inflammatory potential of extract

#### Cell culture

The murine macrophage cell line (RAW264.7 cells) was purchased from American Type Culture Collection (ATCC, CAT #: TIB-71). The cells were maintained in DMEM containing 10% FBS, 0.1% penicillin-streptomycin, 0.1% L-glutamine and 0.1% fungizone at 37°C in a humidified atmosphere containing 5% CO_2_. RAW264.7 cells were subcultured at 3–4 days interval. The cell viability was determined by trypan blue dye exclusion method and direct counting with a hemocytometer.

#### Cell viability

MTT assay was performed as previously described [16] to determine the cytotoxicity effect of *A*. *rugosum* extracts on RAW264.7 cells. Briefly, RAW264.7 cells (4 X 10^3^ cells/well) were seeded on a 96-well plate and incubated overnight at 37°C in a humidified atmosphere containing 5% CO_2_. The attached cells were treated with different concentrations of *A*. *rugosum* extracts. After 24 hours incubation, 5mg/mL of MTT reagent was added to each of the 96 wells. The supernatant was removed after 4 hours incubation and 100% DMSO was added to dissolve the formazan salt. The absorbance was read at 560 nm with a spectrophotometer. The untreated cells incubated in medium only were denoted as a negative control.

#### Nitric oxide determination

The nitric oxide (NO) assay was performed as previously described [16]. RAW264.7 cells (4 X 10^5^ cells/well) were seeded into 96-well plates and incubated at 37°C in a humidified atmosphere containing 5% CO_2_ for 24 hrs. The attached cells were co-incubated with *A*. *rugosum* extract and 1 μg/mL of *Escherichia coli* (O55:B5) lipopolysaccharide (LPS) at 37°C in a humidified atmosphere containing 5% CO_2_ for another 24 hours. Subsequently, 50 μL of culture supernatant was mixed with an equal volume of Griess reagent (1% sulphanilamide and 0.1% N-(1-napthyl)ethylenediamine in 2.5% H_3_PO_4_) and the absorbance was measured at 540 nm. The amount of nitrite in the extract was calculated based on the standard curve generated with sodium nitrite (0–100 μM). The cell viability was determined using MTT assay. L-NAME at a concentration of 250 μM was used as a standard iNOS inhibitor (positive control). Each assay was carried out in triplicate and the results were expressed as micromolar concentration of NO production.

#### Nitric oxide radical scavenging assay

The nitric oxide radical scavenging assay was performed according to the method reported previously [16]. Briefly, 90 μL of sodium nitroprusside (5 mM dissolved in PBS) solution was added to 10 μL of *A*. *rugosum* extract. The plate was incubated for 90 minutes under exposure to light. Next, 100 μL of Griess reagent was added to the wells containing the mixture and the nitrite levels were measured at 540 nm. Quercetin was used as a positive control. Each assay was carried out in triplicate and the results were expressed as percentage of NO production.

#### Immunofluorescence staining of NF-κB p65 and iNOS

RAW264.7 cells (5 X 10^4^ cells/well) were seeded in a chamber slide and incubated at 37°C in a humidified atmosphere containing 5% CO_2_ for overnight. The attached cells were co-incubated with *A*. *rugosum* extract and 1 μg/mL of *Escherichia coli* (O55:B5) lipopolysaccharide (LPS) at 37°C in a humidified atmosphere containing 5% CO_2_ for 24 hours. After the cells were washed with phosphate-buffered saline (PBS), the cells were fixed immediately with 4% of paraformaldehyde for 20 minutes. Then, the cells were permeabilised with 0.5% of PBS-Triton X-100 (PBST) for 10 minutes. The slides were then incubated with primary antibody against NF-κB p65 or iNOS (1: 100 dilution; Cell Signaling Tecnology (CST, USA)) for 2 hours in room temperature. Next, the slides were washed with 0.3% of PBST and incubated with FITC-conjugated anti-rabbit IgG secondary antibodies at 1:80 dilutions (Merck Millipore, Germany) at room temperature for another 2 hours in dark. The slides were then washed again with 0.3% of PBST. The cells were mounted with Prolong^®^ Gold Antifade Reagent with DAPI (Thermo Fisher Scientific, USA). Slides were observed under fluorescence microscope with FITC and DAPI filters and images were captured using Nikon’s Imaging Software, NIS-Elements.

#### Measurement of TNF-α and IL-10 production

The levels of TNF-α and IL-10 were measured using ELISA kits (Thermo Scientific, USA) according to the manufacturer’s instructions. Briefly, RAW264.7 cells (5 X 10^6^ cells) were seeded into a 25 cm^3^ tissue culture flask and incubated at 37°C in a humidified atmosphere containing 5% CO_2_ overnight. The attached cells were co-incubated with *A*. *rugosum* extracts and 1 μg/mL LPS at 37°C in a humidified atmosphere containing 5% CO_2_ for 24 hours. Then, the supernatant were collected to determine the TNF-α and IL-10 levels by ELISA.

#### Gene expression study

RAW264.7 cells (5 X 10^6^ cells) were seeded into a 25 cm^3^ tissue culture flask and incubated at 37°C in a humidified atmosphere containing 5% CO_2_ for 24 hours. The attached cells were co-incubated with *A*. *rugosum* extracts and 1 μg/mL LPS at 37°C in a humidified atmosphere containing 5% CO_2_ for another 24 hours. The RNA extraction was done using RNAqueous^®^-4PCR kit (Ambion, USA). The purity of the isolated RNA was determined based on the ratio of the absorbance at 260 nm and 280 nm. Purified RNA with a A_260_/A_280_ ratio between 1.8 and 2.0 was further used to synthesise complementary DNA (cDNA) by polymerase chain reaction (PCR) approach. The integrity of the RNA samples was assessed using an Agilent^®^ 2100 Bioanalyzer. RNA samples with RNA integrity number (RIN) of 8 to 10 were used for the subsequent experiment. High Capacity cDNA Reverse Transcription Kit (Applied Biosystems, USA) which contains all reagents needed for reverse transcription (RT) of total RNA to single-stranded cDNA was used in this study. Generally, 10 μLHigh Capacity cDNA Reverse Transcription master mix (RT buffer, dNTP mix, randoms primers, Multiscribe reverse^™^ transcriptase enzyme and nuclease free water) was added to 10 μL RNA samples. The mixture was then loaded into a thermal cycler (Biorad, USA) and PCR was carried out according to optimized thermal cycling conditions as recommended by the manufacturer. Table 1 shows the list of genes investigated in this study and the corresponding accession numbers. Mouse β-actin (ACTB) was used as the endogenous control in this study. All TaqMan^®^ probes (Applied Biosystems, USA) used in this investigation were labelled with FAM^™^ reporter dye at the 5’ end and MGB quencher at the 3’ end. The fold change in the target genes normalised to ACTB and relative to LPS was calculated using the 2^-ΔΔCT^ method [18].

**Table 1:** Genes investigated using quantitative reverse transcription PCR (qRT-PCR).

No.	Gene name and abbreviation	Assay ID	Accession number
1	Nuclear factor kappa B (NF-κB) p65	Mm00501346_m1	NM_009045.4
2	Inducible nitric oxide synthase (iNOS)	Mm 00440502_m1	NM_010927.3
3	Tumour necrosis factor alpha (TNF-α)	Mm 00443258_m1	NM_013693.2
4	Interleukin 10 (IL-10)	Mm 00439614_m1	NM_010548.2

General abbreviation of genes selected for this study and corresponding assay ID and accession number were obtained from the Applied Biosystems website and NCBI database. Assay ID refers to the Applied Biosystems Gene Expression Assays inventoried kits with proprietary primer and TaqMan^®^ probe mix. Assay ID with “Mm” is referred to as *“Mus musculus”*. All Gene Expression Assay kits indicated are FAM/MGB probed.

### Antioxidant activity

The DPPH assay was performed as described previously [16]. Five microliters of *A*. *rugosum* extracts were added into a 195 μL reaction mixture which consisted of DPPH radical in ethanol solution. The mixture was incubated for 3 hours in the dark and the absorbance was measured at 515 nm using a spectrophotometer (BioTek, USA). Ascorbic acid, trolox and BHT served as positive controls. The values were expressed as EC_50_. EC_50_ is defined as the amount of antioxidant required to scavenge 50% of the DPPH radicals.

The ABTS assay was used to measure the antioxidant capacity of the *A*. *rugosum* extracts. This assay was carried out based on the method reported previously [16]. Briefly, 7 mM ABTS was mixed with 2.45 mM potassium persulphate and incubated in the dark at room temperature for 12–16 hours to allow the production of ABTS radical cation (ABTS^·+^). The ABTS^·+^ solution was further diluted with ethanol to an absorbance of 0.70 (± 0.02) at 734 nm and equilibrated at 30°C. Then, 100 μL of ABTS^·+^ solution was added to 10 μL of *A*. *rugosum* extract and the absorbance at 734 nm was measured after 1 minute. Ascorbic acid, trolox and BHT were used as positive controls. The values were expressed as EC_50_.

### Total phenolic content (TPC)

Total phenolic content of *A*. *rugosum* extracts were measured by Folin-Ciocalteu’s phenol reagent according to the method reported previously [16]. First, 50 μL of 10% Folin-Ciocalteau phenol reagent was added to 50 μL of extract. The mixed solution was incubated in the dark at room temperature for 3 minutes. Then, 100 μL of 10% Na_2_CO_3_ was added to the solution and was incubated in the dark at room temperature for 1 hr. The absorbance was measured at 750 nm using spectrophotometer. Gallic acid was used as a standard phenolic compound. All samples were analysed in triplicate and the total phenolics were expressed as gallic acid equivalents (GAEs).

### Statistical analysis

All values are expressed as means ± standard deviation (SD) of triplicate values. Statistical analysis was performed using one-way analysis of variance (ANOVA) followed by Duncan’s Multiple Comparison Test using Statistical Product and Service Solutions, SPSS^®^ Statistics for Windows, Version 17.0. Differences were considered statistically significant when *P*< 0.05 and exact *P* values were shown unless *P*< 0.001. Effective concentrations (EC_50_) were calculated using GraphPad Prism software version 5.0.

## Results

### Growth stages

**Fig 1:**
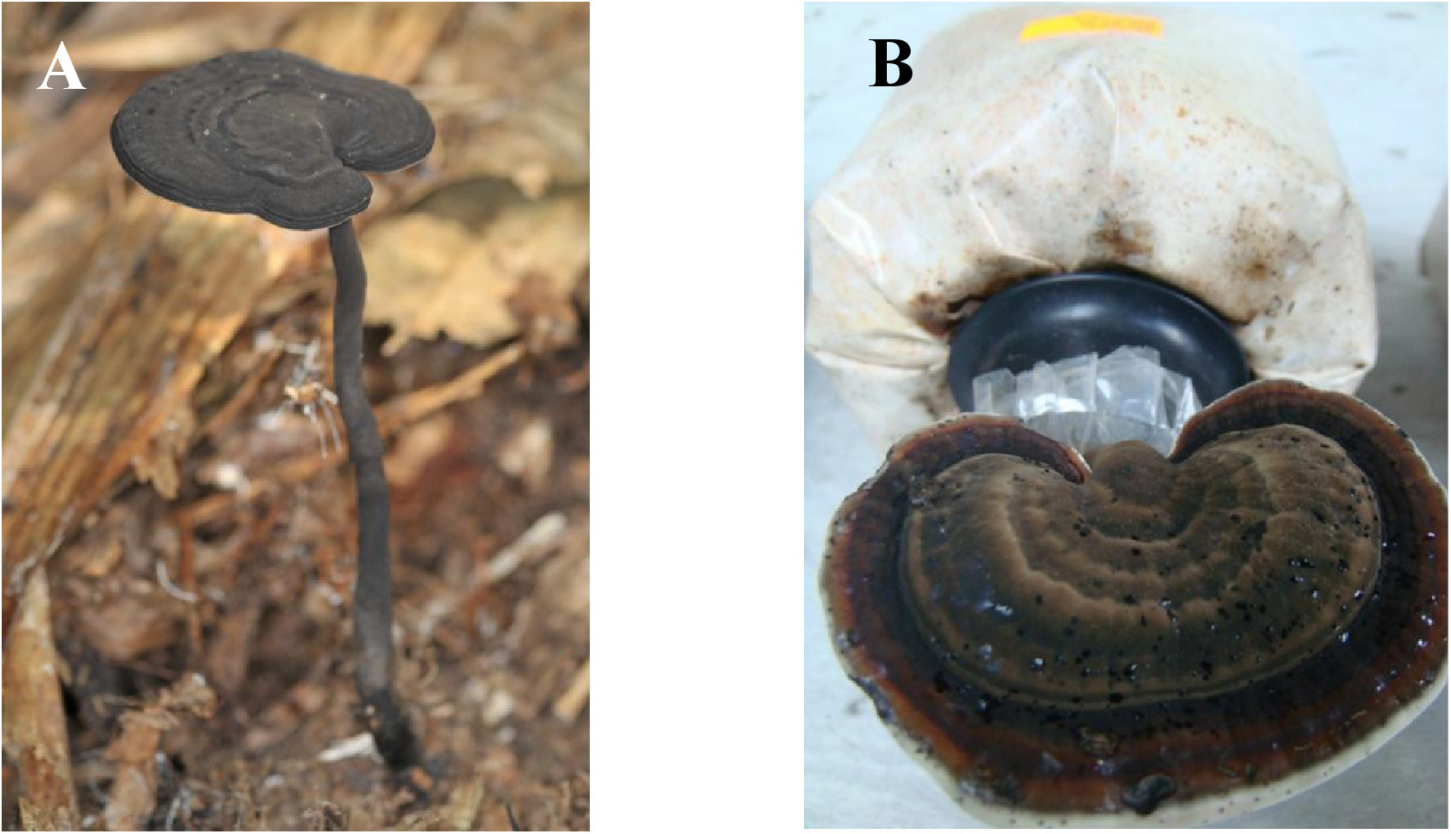
(A) Wild basidiocarp and (B) domesticated basidiocarp of *A*. *rugosum*.

The observations on growth stages of *A*. *rugosum* basidiocarps are depicted in Table 2. The mycelia fully colonised the 300 g substrate in 15 days. In about 25 days, pinhead started emerging and they grew into matured basidiocarps after 1–2 weeks. The domesticated basidiocarps of *A*. *rugosum* (9.6 ± 3.8 g; Fig 1B) showed a higher average fresh weight compared to the basidiocarps grown in the wild (7.6 ± 3.9 g; Fig 1A).

**Table 2:** The colonisation of sawdust bags and basidiocarp formation by *A*. *rugosum*.

Growth stages	DB
Mycelia colonisation (days)	15
Mycelia growth rate (cm / 2 days)	1.9 ± 0.1
Pinhead emergence (days)	25.7 ± 3.5
Fresh weight (g)	9.6 ± 3.8
Biological efficiency (%)*	5.4 ± 2.2

*Based on one harvest; results are expressed as mean ± SD (*n* = 3).

### Effects of WB and DB on RAW264.7 cell viability

**Fig 2:**
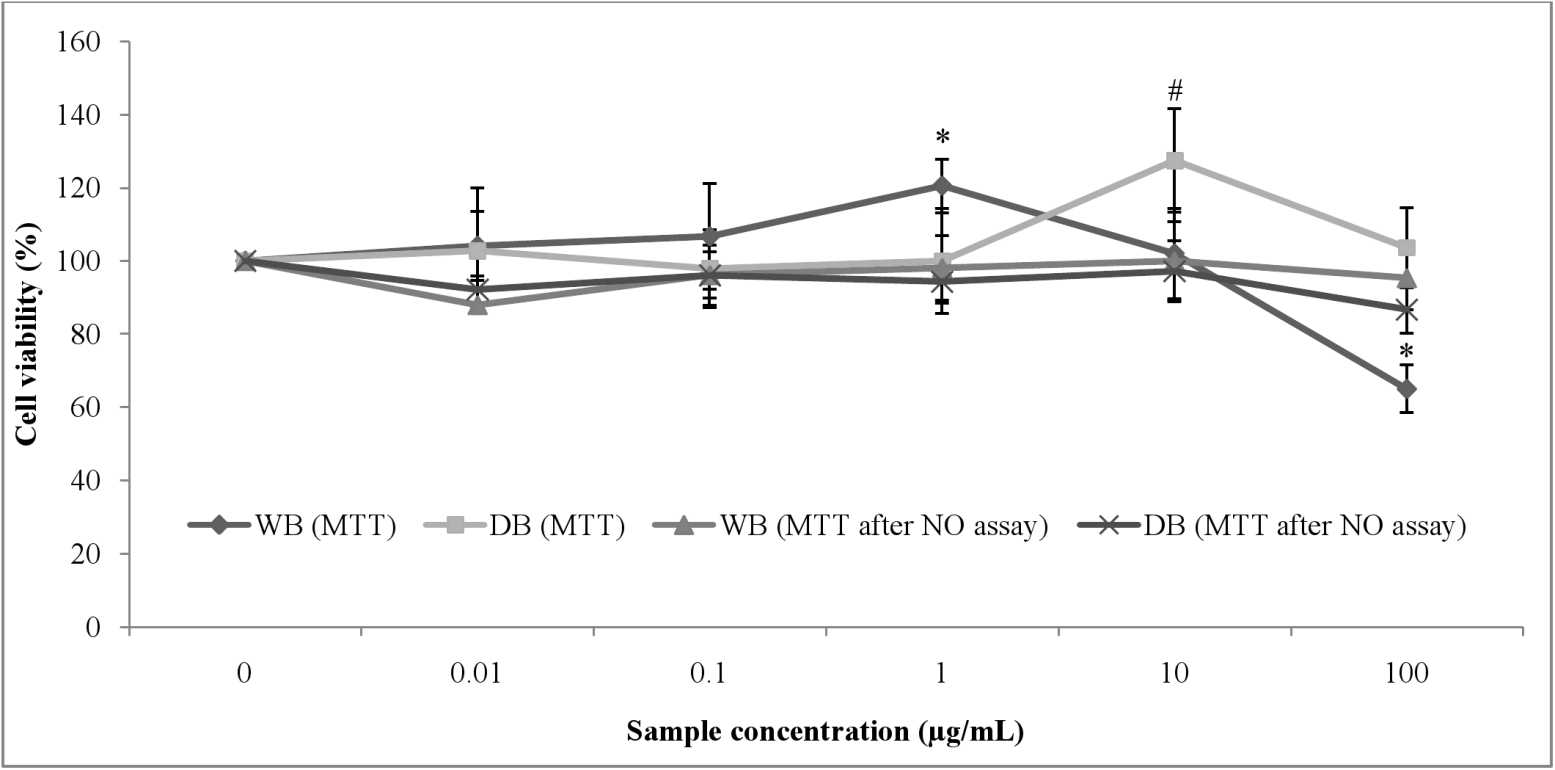
The effects of *A*. *rugosum* extracts on RAW264.7 and LPS-stimulated RAW264.7 cell viability (S1 Dataset). RAW264.7 cells were treated with various concentrations of *A*. *rugosum* extracts. Besides that, cell viability of LPS-stimulated RAW264.7 cells were also assessed using MTT method.The results shown represent the mean ± SEM, n = 3, and ^*^p < 0.05 compared to negative control (untreated cells or LPS-stimulated cells only) denoted as 100%.

MTT assay was performed to examine the cytotoxic effect of *A*. *rugosum* to RAW264.7 cells. The viability of untreated cells (positive control) was denoted as 100%. When RAW264.7 cells were incubated with 0.1, 1, and 10 μg/mL of WB, the cell proliferation was increased by 10.57%, 21.31%, and 21.21%, respectively (Fig 2). However, in the presence of 100 μg/mL of WB, the cell viability decreased by 22.66%.

### Effects of WB and DB on LPS-stimulated NO production and expression

**Fig 3:**
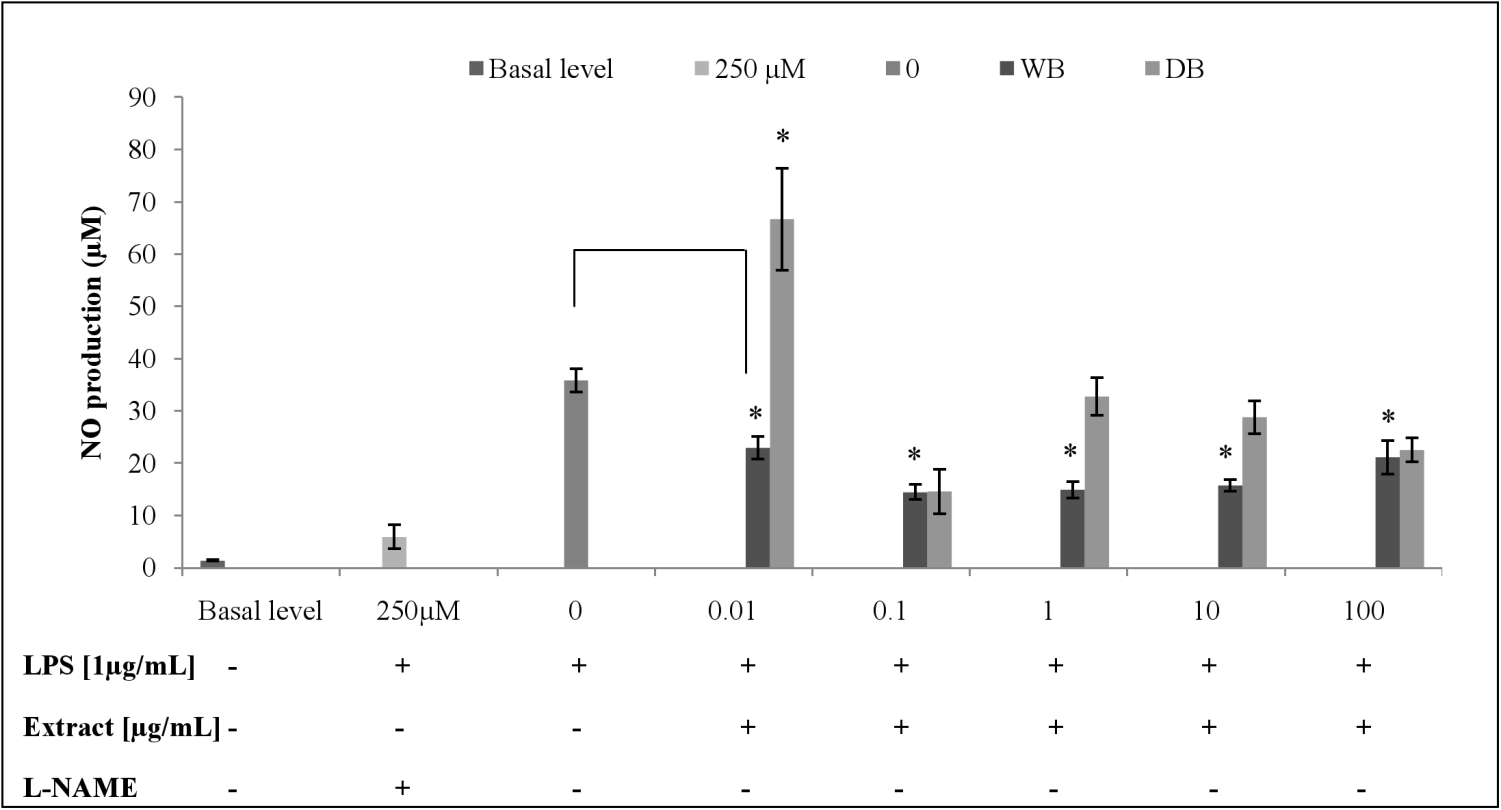
Effects of *A*. *rugosum* extracts on LPS-induced NO production by RAW264.7 cells (S1 Dataset). RAW264.7 cells were co-incubated with various concentrations of *A*. *rugosum* extracts and 1 μg/mL LPS for 24 hrs. L-NAME (250 μM) served as the positive control. Results shown represent the mean ± SD, n = 3 and ^*^p < 0.05 versus LPS-induced NO level alone.

**Fig 4:**
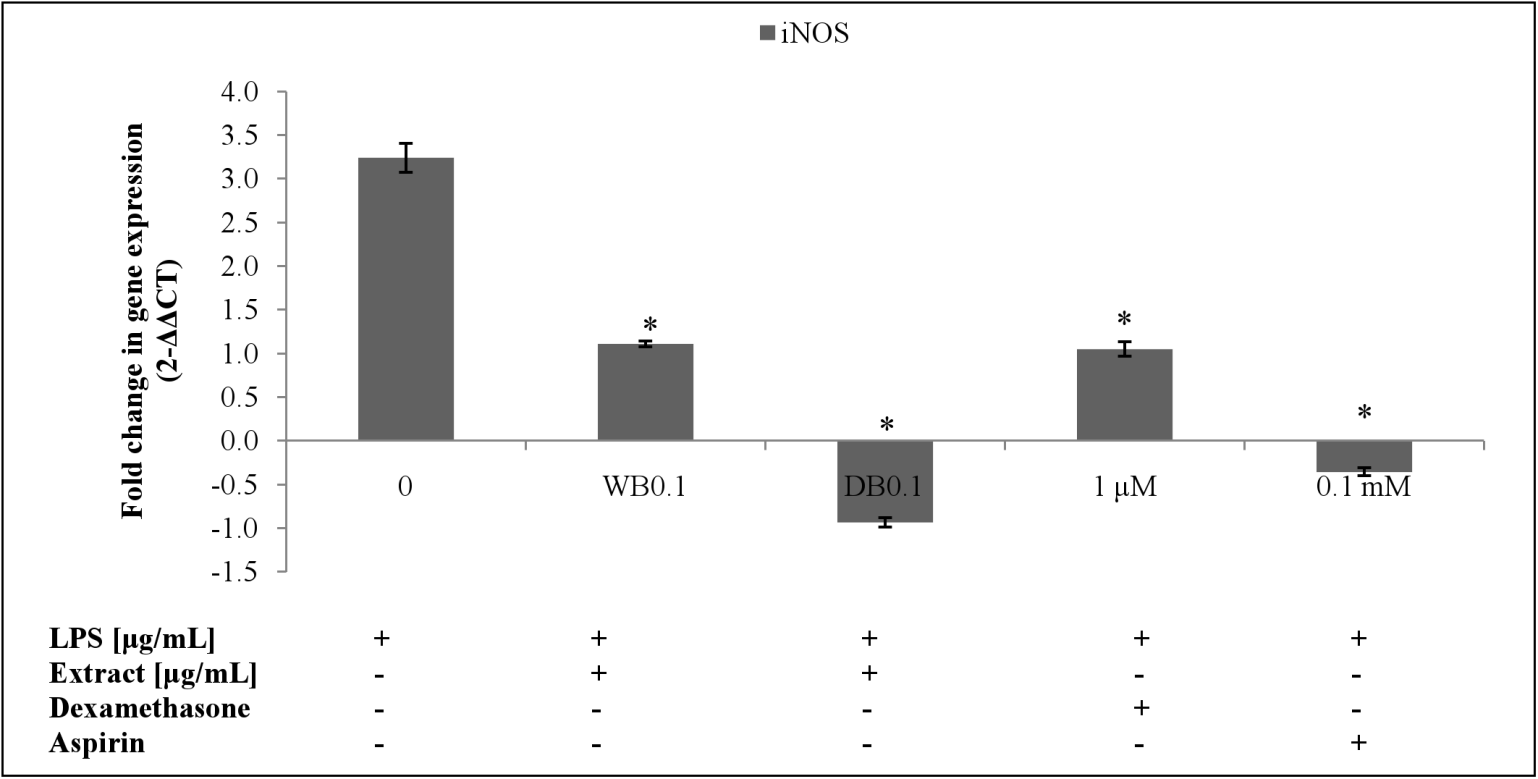
Effects of *A*. *rugosum* extracts on iNOS gene expression (S1 Dataset). RAW264.7 cells were co-incubated with *A*. *rugosum* extracts (WB0.1: 0.1 μg/mL WB; DB0.1: 0.1 μg/mL DB) and 1 μg/mL LPS for 24 hrs. Dexamethasone (1 μM) and aspirin (0.1 mM) served as positive controls. β-actin was used as loading control and normalised against the treated group. Results shown represent the mean ± SEM, n = 3 and ^*^p < 0.05 versus LPS-induced NO level alone.

**Fig 5:**
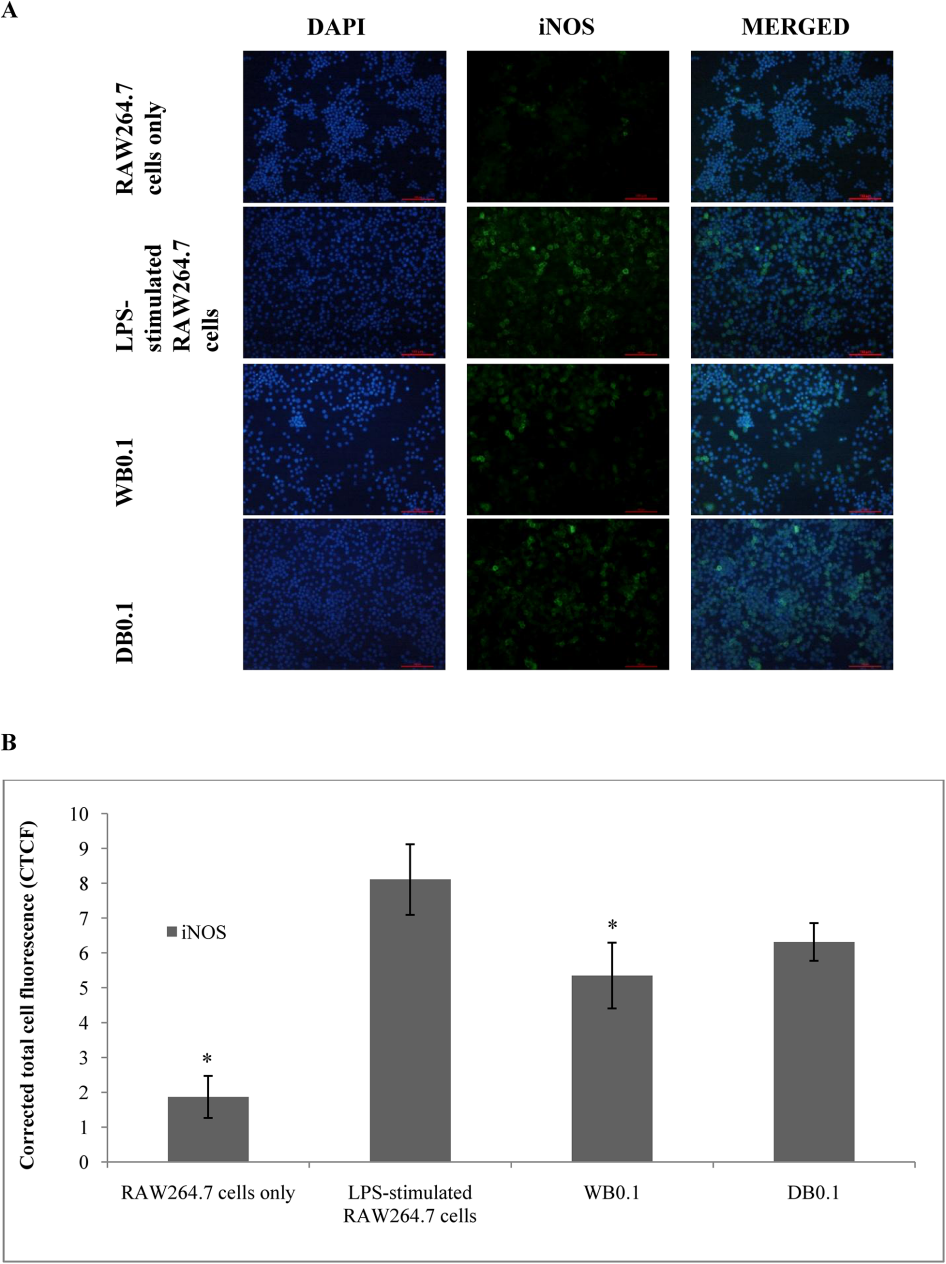
(A) Effects of HF, WB, and DB on iNOS activity in LPS-stimulated RAW264.7 cells. iNOS was localised by fluorescence microscopy after immunofluorescence staining with iNOS antibody (green). Cells were stained with DAPI for visualization of nuclei (blue); (B) Quantification of fluorescence intensity (S1 Dataset). The fluorescence intensity was quantified using ImageJ and displayed in corrected total cell fluorescence (CTCF) Results shown represent the mean ± SD, n = 3 and *p < 0.05 versus LPS-induced iNOS expression alone.

The inhibitory effect of WB and DB on LPS-stimulated NO production in RAW264.7 cells were evaluated using the Griess reaction. Both WB and DB showed inhibition of NO production in LPS-stimulated RAW264.7 cells (Fig 3). At the concentration of 0.1 μg/mL, WB and DB were able to suppress the NO production in LPS-stimulated RAW264.7 cells by 46.79% (14.13 ± 0.69 μM) and 49.03% (13.53 ± 2.07 μM), respectively. L-NAME, a standard NOS inhibitor, used as a positive control in this study significantly (p < 0.001) inhibited NO production (5.91 ± 1.31 μM, 83.50% inhibition) at 250 μM. Fig 2 shows that the inhibitory actions of WB and DB on NO production were not due to cell death. Besides that, the inducible nitric oxide synthase (iNOS) gene in RAW264.7 cells was up-regulated upon stimulation with 1 μg/mL of LPS and was significantly down-regulated with the treatment of WB and DB at the concentration of 0.1 μg/mL (Figs 4, 5A and 5B).

### Effects of WB and DB on NO radical scavenging activity

**Fig 6:**
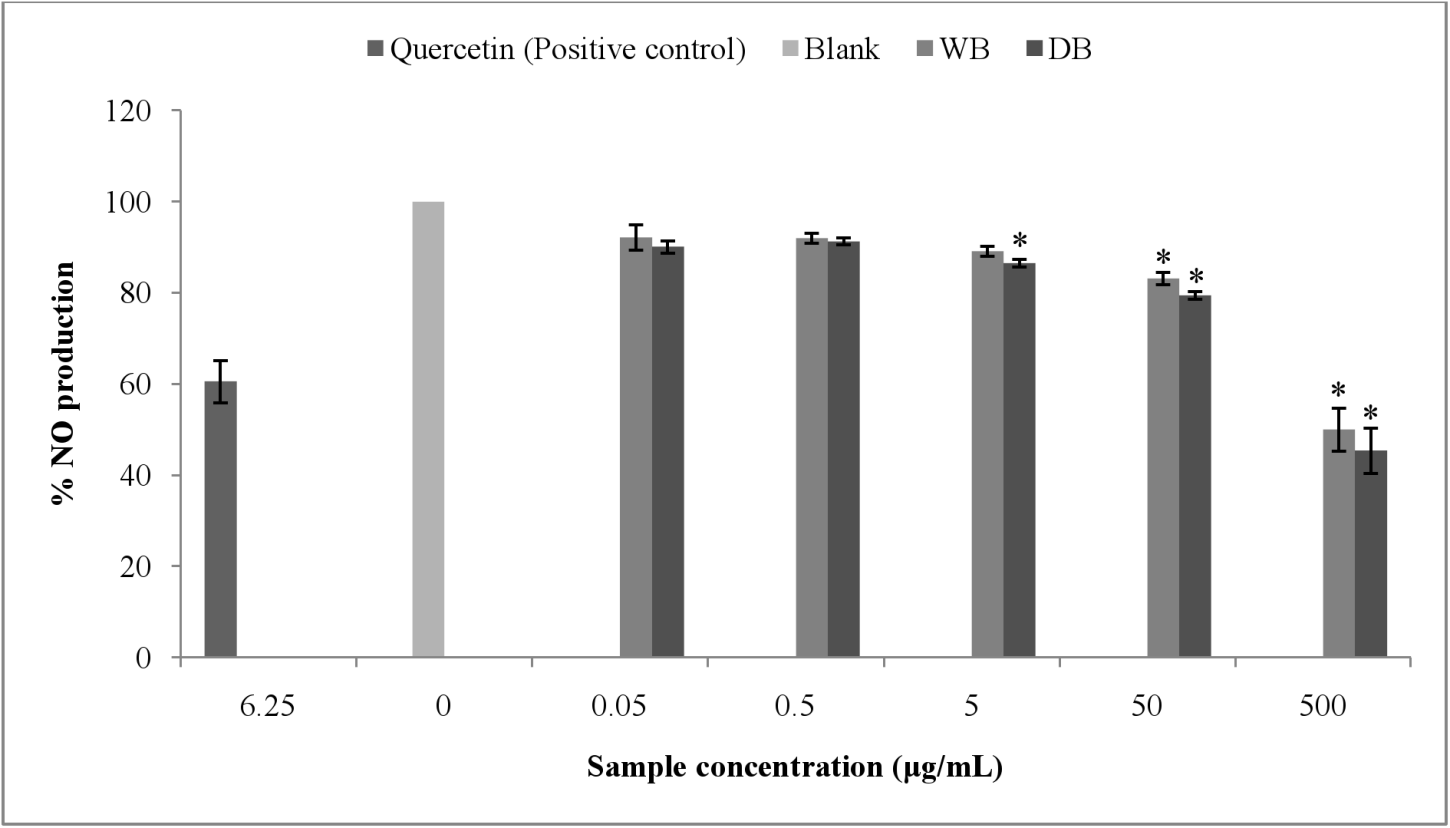
Effects of *A*. *rugosum* extracts on NO production by sodium nitroprusside (SNP) (S1 Dataset). *A*. *rugosum* extracts were co-incubated with SNP (5mM dissolved in PBS) solution for 90 minutes. Quercetin (6.25 μg/mL) was used as the positive control. Results shown represent the mean ± SEM, n = 3 and ^*^p < 0.05 versus SNP-induced NO alone.

The NO scavenging effect of WB and DB were assessed. Both WB and DB scavenged the NO radical in a dose-dependent manner (Fig 6). Significant (p < 0.001) scavenging activities of WB (49.97%) and DB (54.59%) were observed at 500 μg/mL. Quercetin, a flavonoid widely found in plants and well known for its antioxidant and radical scavenging properties was used as a positive control and it scavenged 39.44% of NO radical at 6.25 μg/mL (p < 0.001).

### Effects of WB and DB on LPS-stimulated TNF-α and IL-10 levels and expression in RAW264.7 cells

**Fig 7:**
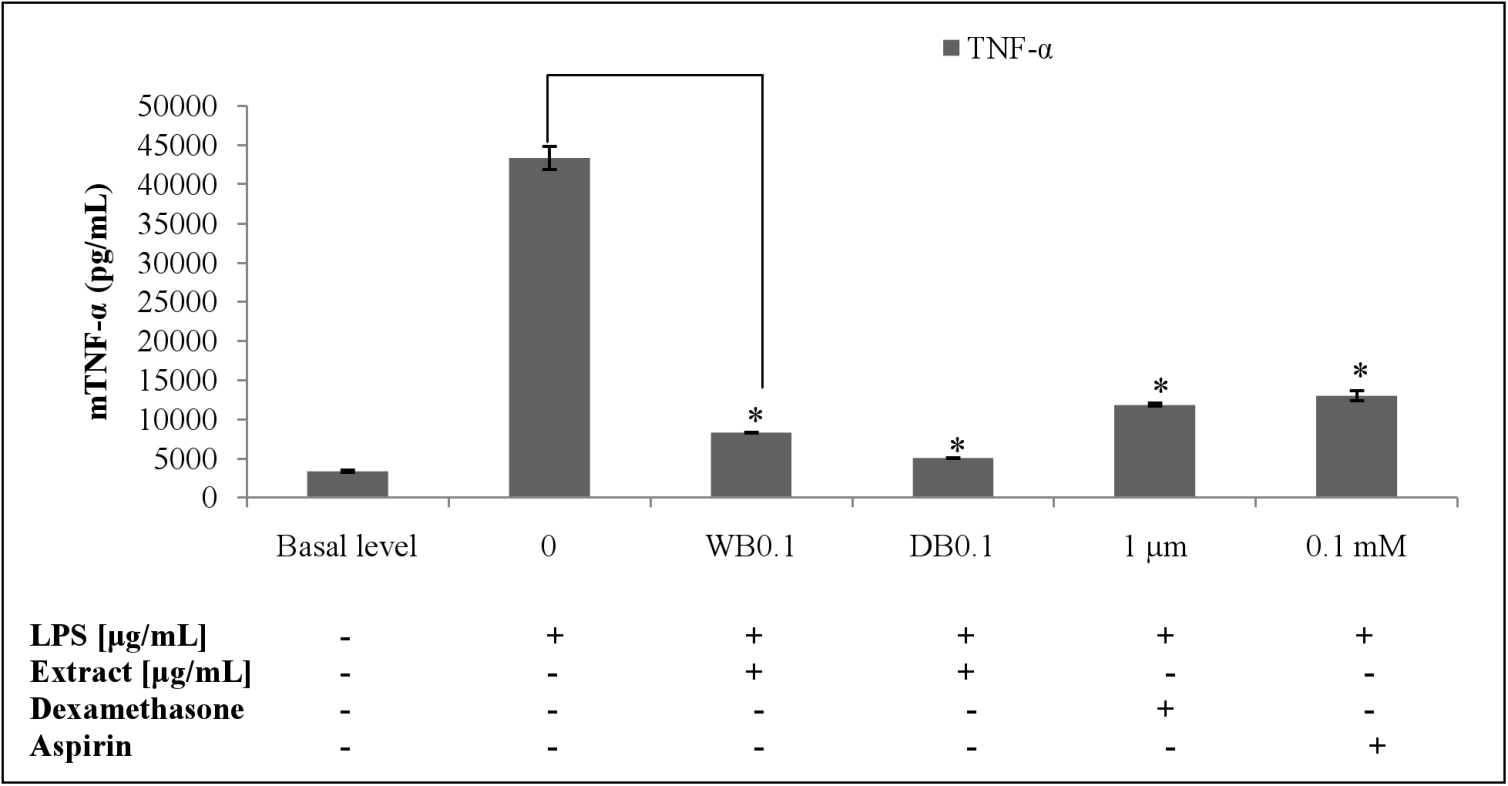
Effects of *A*. *rugosum* extracts on TNF-α cytokine level (S1 Dataset). RAW264.7 cells were co-incubated with *A*. *rugosum* extracts (WB0.1: 0.1 μg/mL WB; DB0.1: 0.1 μg/mL DB) and 1 μg/mL LPS for 24 hrs. Dexamethasone (1 μM) and aspirin (0.1 mM) served as positive controls. Results shown represent the mean ± SD, n = 3 and ^*^p < 0.05 versus LPS-induced NO level alone.

**Fig 8:**
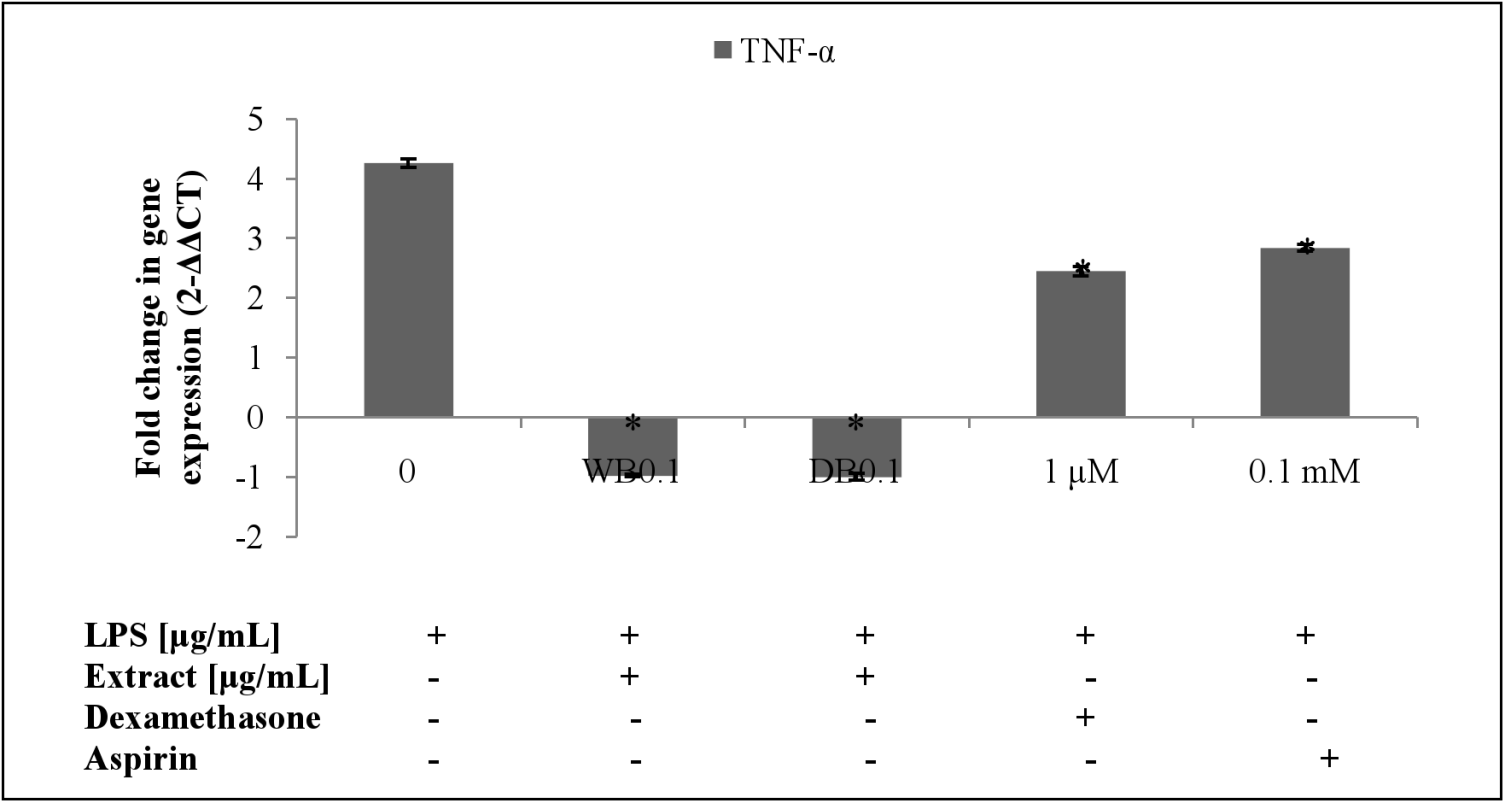
Effects of *A*. *rugosum* extracts on TNF-α gene expression (S1 Dataset). RAW264.7 cells were co-incubated with *A*. *rugosum* extracts (WB0.1: 0.1 μg/mL WB; DB0.1: 0.1 μg/mL DB) and 1 μg/mL LPS for 24 hrs. Dexamethasone (1 μM) and aspirin (0.1 mM) served as positive controls. β-actin was used as loading control and normalised against the treated group. Results shown represent the mean ± SD, n = 3 and ^*^p < 0.05 versus LPS-induced NO level alone.

**Fig 9:**
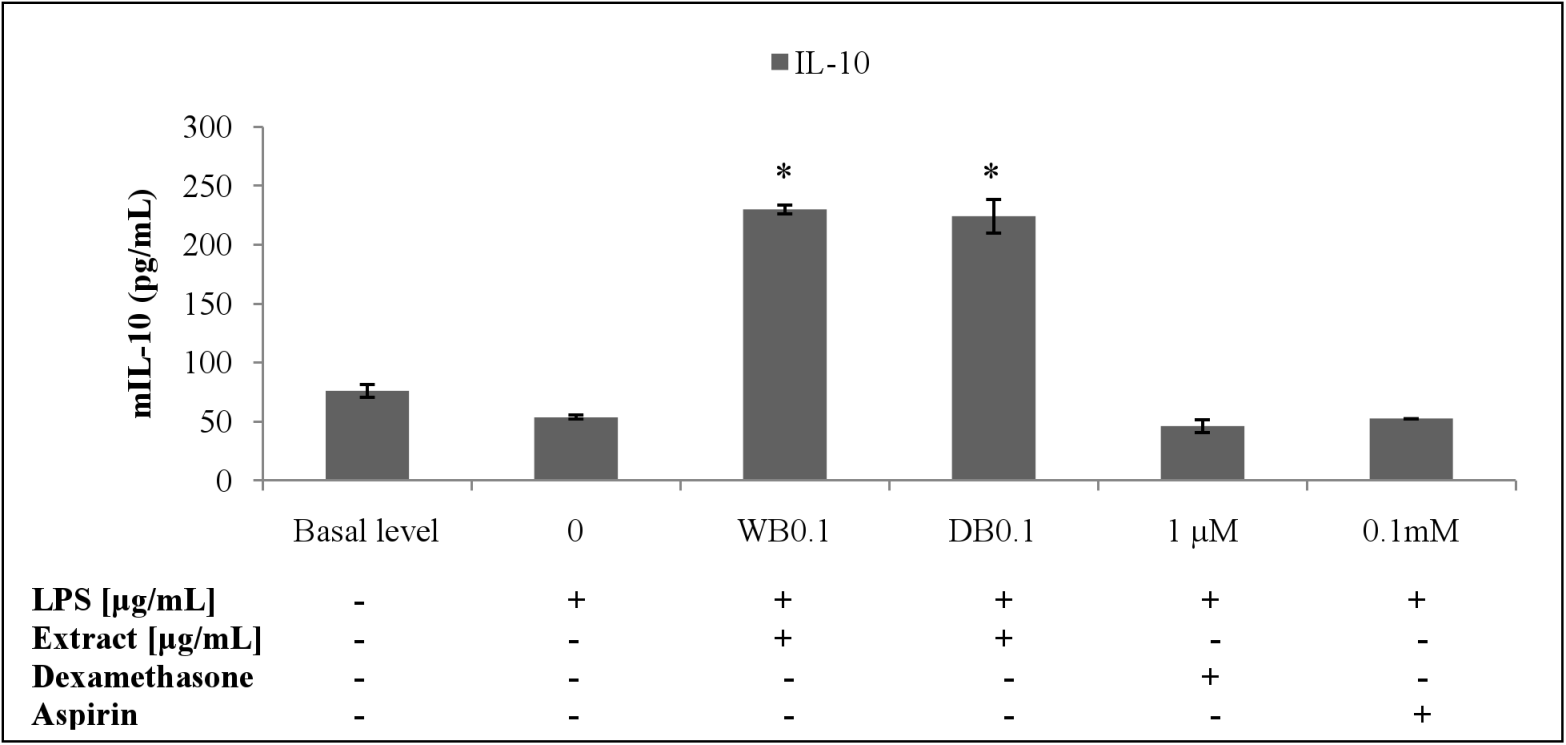
Effects of *A*. *rugosum* extracts on IL-10 cytokine level (S1 Dataset). RAW264.7 cells were co-incubated with *A*. *rugosum* extracts (WB0.1: 0.1 μg/mL WB; DB0.1: 0.1 μg/mL DB) and 1 μg/mL LPS for 24 hrs. Dexamethasone (1 μM) and aspirin (0.1 mM) served as positive controls. Results shown represent the mean ± SD, n = 3 and ^*^p < 0.05 versus LPS-induced NO level alone.

**Fig 10:**
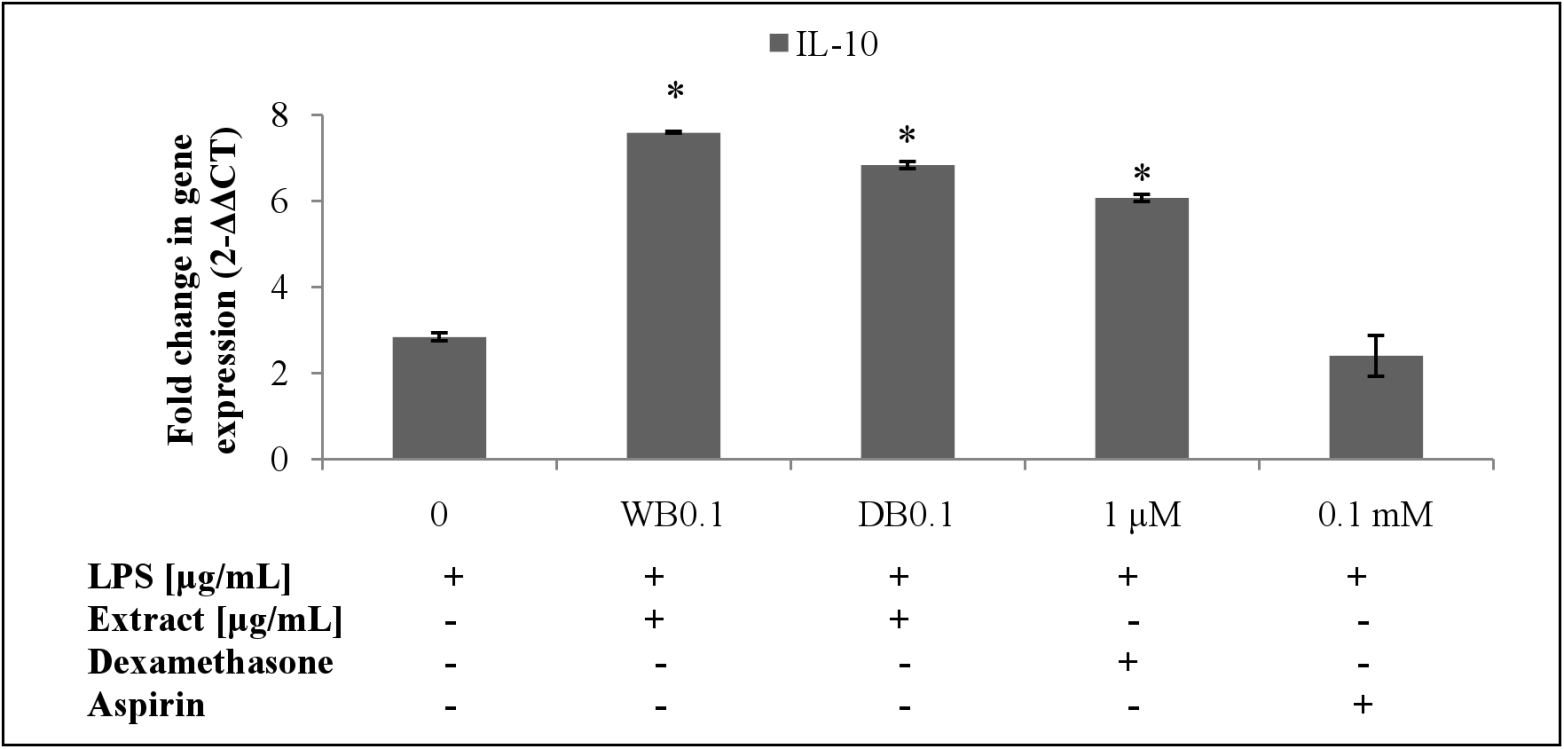
Effects of *A*. *rugosum* extracts on IL-10 gene expression (S1 Dataset). RAW264.7 cells were co-incubated with *A*. *rugosum* extracts (WB0.1: 0.1 μg/mL WB; DB0.1: 0.1 μg/mL DB) and 1 μg/mL LPS for 24 hrs. Dexamethasone (1 μM) and aspirin (0.1 mM) served as positive controls. β-actin was used as loading control and normalised against the treated group. Results shown represent the mean ± SD, n = 3 and ^*^p < 0.05 versus LPS-induced NO level alone.

The WB and DB attenuated the level of TNF-α in LPS-stimulated RAW264.7 cells (Fig 7) which was due to the down-regulation of the TNF-α gene expression (Fig 8). Besides, WB and DB promoted the production of IL-10, an anti-inflammatory cytokine (Fig 9). Significant up-regulation of IL-10 expression by WB and DB was evident (Fig 10).

### Effects of WB and DB on LPS-stimulated NF-κB p65 expression in RAW264.7

**Fig 11:**
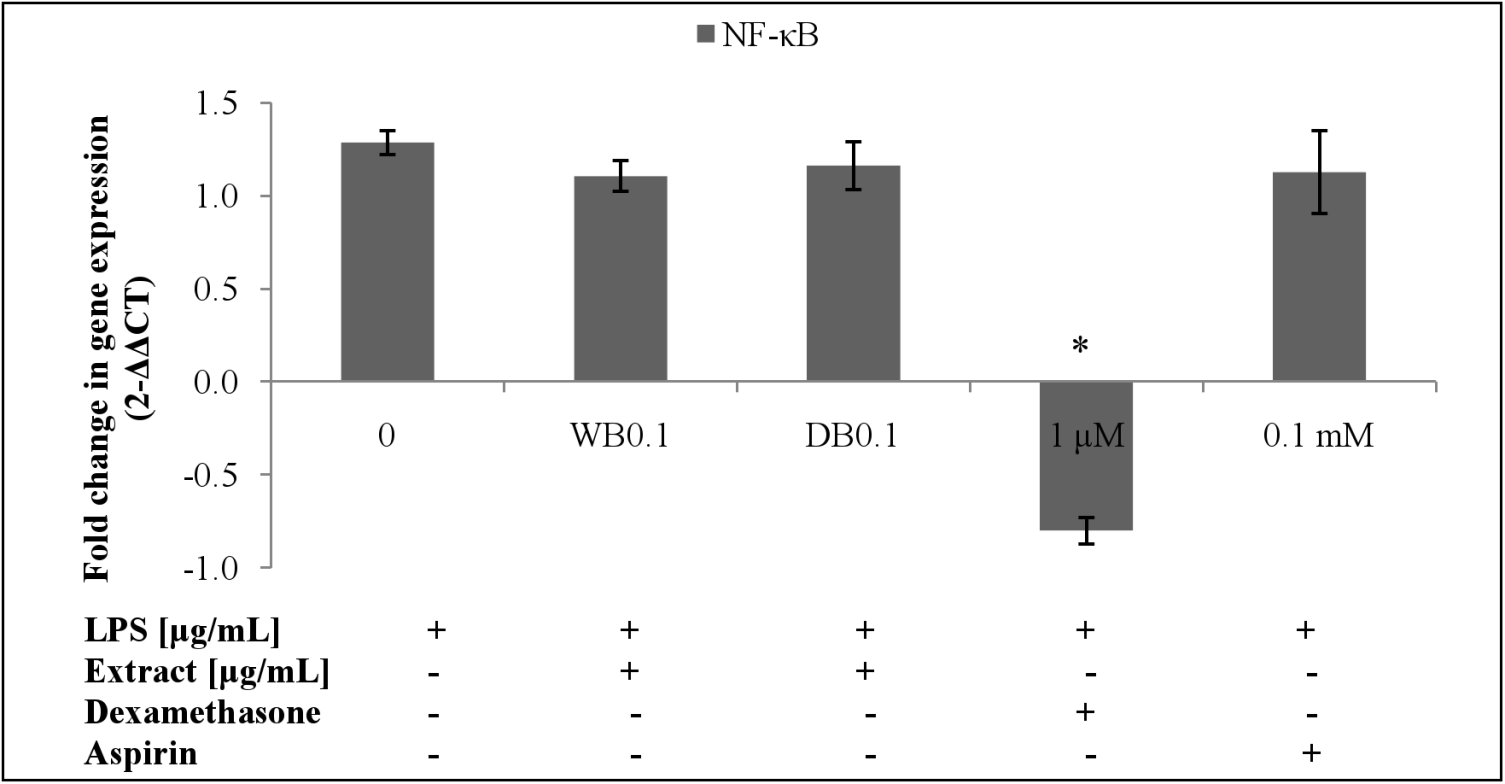
Effects of *A*. *rugosum* extracts on NF-κB p65 gene expression (S1 Dataset). RAW264.7 cells were co-incubated with *A*. *rugosum* extracts (WB0.1: 0.1 μg/mL WB; DB0.1: 0.1 μg/mL DB) and 1 μg/mL LPS for 24 hrs. Dexamethasone (1 μM) and aspirin (0.1 mM) served as positive controls. β-actin was used as loading control and normalised against the treated group. Results shown represent the mean ± SD, n = 3 and ^*^p < 0.05 versus LPS-induced NO level alone.

**Fig 12:**
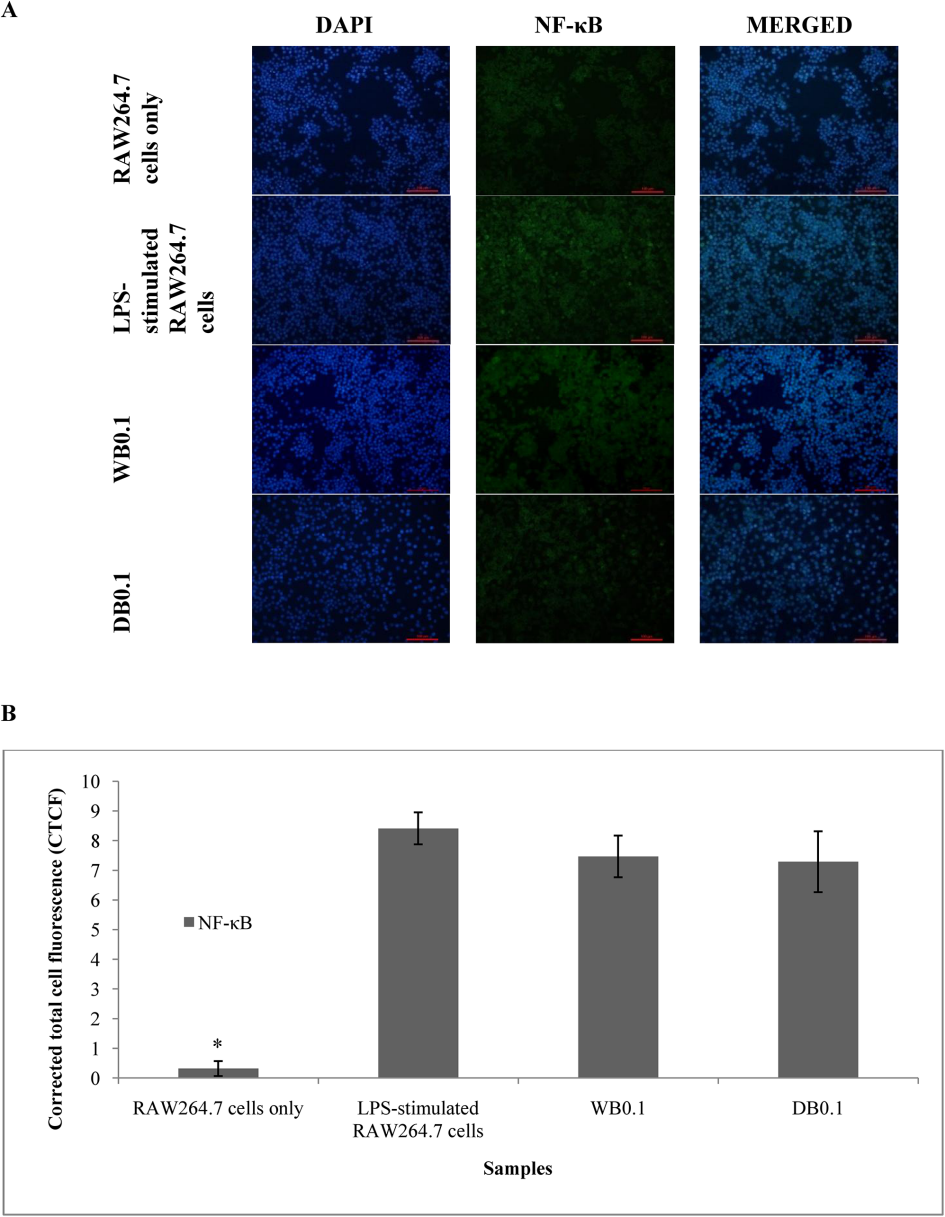
(A) Effects of *A*. *rugosum* extracts on NF-κB activity in LPS-stimulated RAW264.7 cells. NF-κB p65 was localised by fluorescence microscopy after immunofluorescence staining with NF-κB p65 antibody (green). Cells were stained with DAPI for visualization of nuclei (blue); (B) Quantification of fluorescence intensity (S1 Dataset). The fluorescence intensity was quantified using ImageJ and displayed in CTCF. Results shown represent the mean ± SD, n = 3 and *p < 0.05 versus LPS-induced p65 NF-κB expression alone.

LPS-stimulated proinflammatory mediators production in RAW264.7 cells have been reported to be induced through NF-κB activation [19]. Fig 11 shows the effect of WB and DB on the NF-κB p65 gene expression level in LPS-stimulated RAW264.7 cells. There was no significant effect of WB and DB on the NF-κB p65 subunit. Protein expression showed presence of NF-κB p65 activity (Fig 12A and 12B). Dexamethasone (1 μM) and aspirin (0.1 mM) were used as positive controls.

### Antioxidant activity and total phenolic content

Table 3 shows the antioxidant activity and total phenolic content of WB and DB. The antioxidant activity of *A*. *rugosum* extracts were evaluated based on DPPH and ABTS assays. Both WB and DB showed similar total phenolic content and DPPH scavenging activity. However, WB displayed a higher ABTS scavenging activity compared to DB.

**Table 3:** Antioxidant activity and total phenolic content of WB and DB.

	Total phenolic content (mg of GAEs / g of extract)	DPPH (EC_50_μg/mL)	ABTS (EC_50_μg/mL)
Ascorbic acid	-	2.41	9.68
BHT	-	6.30	22.37
Trolox	-	3.88	16.63
WB	47.89 ± 0.01	76.86	222.90
DB	46.39 ± 0.01	73.28	469.60

DPPH and ABTS are expressed as half maximal effective concentrations (EC_50_); TPC results are expressed as mean ± SD (*n* = 3).

## Discussion

Mushroom cultivation is a profitable agribusiness. The development and innovation in mushroom cultivation is important for food security, reduction of poverty, and development of economic status of small farmers [20]. Mushrooms are sources of food and contain useful medicinal properties against diabetes [21], cancer [22], and heart disorder [23]. *Amauroderma rugosum* has been documented to be traditionally used as a remedy to prevent epileptic episodes in children [14], [15]. Clinical and experimental evidences suggest that infection or inflammation may be the main contributor to seizure predisposition and occurrence, and other neurological disorders such as epilepsy [24]. It has also been reported that proinflammatory cytokines are involved in the pathophysiology of seizures and may be a new target for therapies against epilepsy [25–27]. Besides, the Chinese population has been using *A*. *rugosum* to reduce inflammation, to treat diuretic and upset stomach, and it is believed to have cancer prevention properties [13]. To our knowledge, the antioxidant and anti-inflammatory properties of *A*. *rugosum* are yet to be explored and this is the first report which shows successful cultivation of *A*. *rugosum*. The mycelium of *A*. *rugosum* was obtained from tissue culture and maintained on PDA medium. Overall, the growth rate of *A*. *rugosum* was fast as compared to the other mushrooms reported previously (Table 4). Studies have shown that factors such as genotype of mushroom strains, type of substrate, and environment susceptibility, may affect the growth and fructification of mushrooms [17, 28].

**Table 4:** Comparison of spawn run and yield of selected domesticated or cultivated mushrooms.

Mushroom	Substrate	Bag size (g)	Spawn run (Days)	Pinhead emergence (Days)	No. of harvest	Biological efficiency (%)
*A*. *rugosum* (This study)	Sawdust, rice bran	300	15	26	1	5.4
*Oudemansiella tanzanica* [29]	Sawdust, rice bran,	1000	19	2	20	101.9
*Volvariella spp*. [30]	50% Wheat, 50% rice bran	-	12	6	3	13.6
*Pleurotus ostreatus* [31]	Paddy	800	32	50	5	12.0
*Pleurotus ostreatus* [31]	Sorghum	800	23	36	5	25.4
*Pleurotus ostreatus* [31]	Kurakkan	800	21	35	5	30.8
*Pleurotus ostreatus* [31]	Maize	800	22	45	5	16.6
*Pleurotus ostreatus* [20]	Sawdust, wheat straw	1000	17	25	11	43.6
*Pleurotus ostreatus* [20]	Sawdust	1000	17	24	22	64.7
*Lentinus squarrosulus* [32]	*Spondias mombin*, rice bran	-	28	7	12	10.3

It has generally been known that the overproduction of ROS can adversely alter and damage lipids, proteins and DNA, and have been implicated in a number of human diseases. Thus, extensive research has been actively conducted for many years to search for antioxidant properties in natural products. Recently, mushrooms have gained significant interest as a new source of therapeutic agents. Many studies on antioxidant activity of mushrooms have been conducted [33–36]. In the present study, WB and DB showed similar total phenolic content and DPPH scavenging activity. However, WB displayed a higher ABTS cation radical scavenging activity as compared to DB. Alvarez-Parrilla et al. [37] showed that in general, wild mushrooms have higher phenolic content than commercial ones. The same group of researchers also proved that the methanolic extract of wild *Agaricus bisporus* has higher total phenol concentration than the commercial species. Conversely, the present study showed ethanolic extracts of wild and domesticated *A*. *rugosum* have similar total phenolic content. The discrepancy between the two studies may be due to the difference in the growth conditions and types of polyphenolic components present in the extracts.

The MTT assay was performed to assess the cytotoxic effect of WB and DB on RAW264.7 cells. *A*. *rugosum* extracts showed no toxic effect on RAW264.7 cells except for WB at a concentration of 100 μg/mL. Nitric oxide (NO) is a free radical generated by nitric oxide synthase (NOS) from L-arginine and mediates a variety of biological actions such as vasodilation, neurotransmission, inhibition of platelet adherence and aggregation, and macrophage-and-neutrophil-mediated killing of pathogens. However, excessive production of NO has been implicated in the pathogenesis of a variety of inflammatory diseases [38]. Griess assay was used to measure the accumulation of nitrite, which is the stable metabolite of NO. The quantity of nitrite in the culture medium was measured as an indicator of NO production. The current study showed that the *A*. *rugosum* extracts were able to scavenge NO radicals and suppress the NO production in macrophages. Both WB and DB showed inhibition of NO production in LPS-stimulated RAW264.7 cells at a concentration of 0.1 μg/mL. To determine whether the inhibition of NO production was due to cell death or down-regulation of iNOS expression, the MTT assay was performed after challenging the cells with LPS. The inhibition of NO production in LPS-stimulated macrophages by WB and DB was found to be not due to the reduction in the number of cells. In fact, *A*. *rugosum* extracts markedly down-regulated the expression of iNOS gene, which is responsible for the production of NO. In addition, WB and DB were able to scavenge the NO radicals in a dose-dependent manner and at a concentration of 500 μg/mL, both WB and DB scavenged > 50% of NO radicals. Although WB and DB showed effective NO scavenging activity at 500 μg/mL, this concentration was not used in cell-based bioassay experiments, as concentration of extracts exceeding 100 μg/mL were cytotoxic to macrophage cells. A previous report on plants showed that high level of antioxidants is associated with genotoxic properties in human peripheral blood mononuclear cells [39]. The DB did not exert a dose-dependent effect on NO production in LPS-stimulated RAW264.7 cells. Unexpectedly, at the lowest concentration tested (0.01 μg/mL), DB caused significant elevation of NO production but in contrast, at higher concentrations this extract inhibited NO production effectively in LPS-stimulated RAW264.7 cells. This may in part be attributed to the “double-edged sword” effect that many natural compounds are known to exert on living systems and DB used in the present study is not an exception. One plausible explanation is that, low concentration of extract may promote inflammation as active compounds in the extract may be recognised as foreign substance by macrophages. However, at higher concentrations, the mixture of the compounds present in the extract may work synergistically to facilitate better interactions in the signal transduction pathway to affect a favourable response by inhibiting NO production effectively. This suggests that critical dosage of DB is required to exhibit potent anti-inflammatory effect. In the initial screening of *A*. *rugosum* extracts, WB showed a slight toxic effect towards RAW264.7 cells (4 X 10^3^ cells/well). However, the MTT assay carried out after the removal of spent media for NO assay, WB showed no toxic effect towards LPS-stimulated RAW264.7 cells (4 X 10^5^ cells/well) which possibly due to the significantly larger number of cells. Evidently, the concentration of the extract (based on extract w/v) was not sufficient to exert significant toxicity on the much higher concentration of cells.

Nuclear factor kappa B (NF-κB) is a transcription factor that has been found to be active in immune response, apoptosis and cellular growth [40]. It is tightly regulated by interaction with inhibitory IκB proteins such as IκBα, IκBβ, IκBγ and IκBε [41]. Activation of NF-κB pathway through the binding of stimuli to TLR-4 causes phosphorylation of IκB proteins by IκB kinase (IKK). The phosphorylated IκB followed by its ubiquitination and 26S proteosomal degradation leads to the dissociation of IκB and enables the free NF-κB to translocate into the nucleus and bind to the target genes [42, 43]. The NF-κB regulates a series of gene expression that are responsible for immune responses. These targeted genes include proinflammatory cytokines (e.g. IL-1, IL-2, IL-6, TNF-α), anti-inflammatory cytokines (e.g. IL-10), chemokines (e.g. IL-8), adhesion molecules (e.g. endothelial leukocyte adhesion molecule, vascular cell adhesion molecule and intercellular adhesion molecule), and inducible enzymes (e.g. iNOS and COX-2) [34, 38]. However, overproduction of proinflammatory mediators often leads to fatal outcomes of chronic inflammatory diseases such as arthritis, asthma, multiple sclerosis and atherosclerosis [44, 45, 46]. Therefore, blocking the activity of proinflammatory cytokines and specific inflammatory pathways such as NF-κB may serve as a useful strategy for treating inflammation.

The present study demonstrated that WB and DB possessed a suppressive effect on downstream production of inflammatory mediators of nitric oxide and TNF-α, and promoted the production and expression of anti-inflammatory cytokine IL-10. This is similar to the study done by Jedinak et al. [47], which also demonstrated that the oyster mushroom concentrate markedly inhibited the expression of iNOS and TNF-α in LPS-stimulated macrophages. On the other hand, IL-10 is a potent anti-inflammatory cytokine which controls the inflammatory processes by suppressing various proinflammatory mediators [48]. Numerous studies have suggested that the induction of IL-10 production might exert beneficial effects in reducing the chronic inflammatory response found in patients with inflammatory disorders [49–51]. NF-κB plays a pivotal role in the immune response, where it controls the expression of various inflammatory cytokines. WB and DB did not show any inhibitory effect on the nuclear translocation of NF-κB p65. Therefore, it is possible that attenuation of proinflammatory mediators is attributed to direct inhibition.Alternatively, DB may be effective in inhibiting other upstream inflammatory signalling molecules such as p38 MAPK and JNK signalling [52, 53]. Hence, further studies are required to investigate on the upstream mechanism of WB and DB on inflammatory pathways.

On the whole, WB and DB showed similar total phenolic content and DPPH scavenging activity except for ABTS scavenging activity, WB displayed a better antioxidant activity than DB. It is possible that WB may contain different types of polyphenols which contribute to better ABTS scavenging activity. Besides, ABTS assay is the second rapid and precise method of determining antioxidant activity and it is appropriate for hydrophilic and lipophilic antioxidant systems. On the other hand, DPPH assay has more limitations and lower sensitivity, and it is only applicable for hydrophobic systems [54]. For the evaluation of anti-inflammatory activities, generally DB displayed slightly better anti-inflammatory activities than WB except for nitric oxide, IL-10, and NF-κB activities, both crude ethanolic extracts of wild and domesticated basidiocarps showed comparable results. Domesticated basidiocarps may exhibit better activities than the wild basidiocarps due to the optimised and controlled cultivation medium and growth condition. Also, media constituents and composition may influence fungi and plant cells metabolism and production of metabolites.

## Conclusion

Overall, wild (WB) and domesticated (DB) basidiocarps of *A*. *rugosum* possessed significant antioxidant and anti-inflammatory activities. Both WB and DB had comparable total phenolic content and DPPH scavenging activity, but WB displayed higher ABTS cation scavenging activity compared to DB. Besides, WB and DB were able to scavenge NO radicals and suppress NO production in LPS-stimulated RAW264.7 cells. The suppressive effect was associated with the down-regulation of the iNOS gene. Moreover, WB and DB demonstrated effective down-regulation of the proinflammatory cytokine, TNF-α and up-regulation of anti-inflammatory cytokine, IL-10. However, the nuclear translocation of NF-κB p65 was not blocked. In summary, anti-inflammatory activity of *A*. *rugosum* demonstrated in this study validated the traditional use of this mushroom to reduce inflammation by the Chinese population.

## Supporting Information

S1 Dataset Dataset of MTT assay, NO assay, MTT after NO assay, CTCF, ELISA cytokines of TNF-α and IL-10, and gene expression data of NF-κB, iNOS, TNF-α and IL-10. (XLSX)

## Abbreviations

LPS: lipopolysaccharide
WB: wild basidiocarp
DB: domesticated basidiocarp
DPPH: 2, 2-diphenyl-1-picrylhydrazyl
ABTS: 2,2’-Azino-bis(3-ethylbenzothiazoline-6-sulphonic acid)
NO: nitric oxide
iNOS: inducible nitric oxide synthase
TNF-α: tumour necrosis factor alpha
IL-10: interleukin-10
NF-κB: nuclear factor kappa B
ROS: reactive oxygen species
IL-1β: interleukin-1β
IFN-γ: interferon-γ
TLR-4: toll-like receptor-4
IL-6: interleukin-6
IL-1: interleukin-1
IL-2: interleukin-2
IL-8: interleukin-8
COX-2: cyclooxygenase-2
IκB: inhibitor kappa B
PDA: potato dextrose agar
PDB: potato dextrose broth
CaCO_3_: calcium carbonate
DMSO: dimethyl sulfoxide
BHT: butylated hydroxytoluene
DMEM: Dulbecco’s Modified Eagle’s Medium
FBS: foetal bovine serum
L-NAME: N_ω_-nitro-l-arginine-methyl ester
H_3_PO_4_: phosphoric acid
MTT: 3-(4,5-dimethylthiazol-2-yl)-2,5-diphenyltetrazolium bromide
Na_2_CO_3_: sodium carbonate
PBS: phosphate buffer saline
PBST: phosphate buffer saline-triton X-100
cDNA: complementary DNA
PCR: polymerase chain reaction
ACTB: mouse β-actin
GAEs: gallic acid equivalents
SD: standard deviation
ANOVA: analysis of variance
EC_50_: effective concentrations
